# Effect of Aerobic Exercise on CD4 Cell Count and Lipid Profile of HIV Infected Persons in North Eastern Nigeria

**DOI:** 10.4172/2155-6113.1000508

**Published:** 2015-10-19

**Authors:** Maduagwu SM, Kaidal A, Gashau W, Balami A, Ojiakor AC, Denue BA, Kida I

**Affiliations:** 1 Department of Physiotherapy, University of Maiduguri Teaching Hospital, Maiduguri, Borno State, Nigeria; 2 Department of Physical and Health Education, University of Maiduguri, Maiduguri, Borno State, Nigeria; 3 College of Medical Sciences, University of Maiduguri, Maiduguri, Borno State, Nigeria; 4 Department of Nursing Services, University of Maiduguri Teaching Hospital, Maiduguri, Borno State

**Keywords:** HIV infected persons, CD4 cell counts, Lipid profile, Antiretroviral clinic, Moderate intensity treadmill aerobic exercise, Lectures on nutrition, Attrition rate

## Abstract

**Background:**

Literature consistently shows dearth of published data from developing countries on effect of exercise on HIV infected persons.

**Objective:**

The study was aimed at determining effect of aerobic exercise on CD4 cell counts and lipid profile of HIV infected persons in Northeastern Nigeria.

**Methods:**

Sample of convenience was employed to enroll volunteer and willing 91 HIV infected persons attending antiretroviral clinic at a tertiary hospital in Northeastern Nigeria. Eighty two met the inclusion criteria and participated in the study. Participants were randomly assigned to experimental and control groups. Baseline values of the variables were determined. Experimental group participated in moderate intensity treadmill aerobic exercise for 12 weeks. Control group participated in weekly lectures on nutrition, adherence to therapy among others. At the end, the study recorded 22% attrition rate, leaving 32 participants in each group (64 participants in both). After the 12 weeks, the variables were re-evaluated. Descriptive statistic summarized the socio-demographic characteristics of the participants. Paired and unpaired Student t-tests analyzed the significant difference in mean values of the variables.

**Results:**

Mean ages in years of the 64 participants, the control and experimental groups were 39.57 ± 10.13, 39.38 ± 10.03 and 40.84 ± 10.05 respectively. There was significant improvement (p < 0.05) in the variables between pre- and post-tests in the experimental group. In the control group, there was either no significant change (p > 0.05) or significant deterioration (p < 0.05) in lipid profile between pre- and post-tests, while in CD4 cell counts, significant improvement was observed. Significant difference (p < 0.05) existed in the variables at the end of the study between both groups.

**Conclusion:**

CD4 cell counts and lipid profile of HIV infected persons who participated in the 12 weeks moderate intensity treadmill aerobic exercise significantly improved. Proper nutrition and adherence to antiretroviral therapy may enhance immune function in HIV population.

## Introduction

It is generally believed that since the evolution of human race, no disease, apart from the early 20th century Spanish flu as reported by Smith [1], and the recent Ebola virus, has generated so many ripples in the world than the human immunodeficiency virus (HIV) pandemic with enormous resources being dispensed to curb its menace. HIV infected persons present with several physiological disturbances, alteration in lipid profile being one of such [2]. This population also experiences diminution in immune function portrayed by reduction in cluster of differentiation 4 (CD4) cells [2]. These cells are selectively attacked by HIV because they possess specific surface receptors that facilitate cellular fusion with HIV glycoprotein complex [3].

With advent of highly active antiretroviral therapy (HAART) about three decades ago, HIV infection changed from a fatal condition to a manageable chronic illness. This transformation is as a result of HAART’s’ ability to inhibit the growth and replication of HIV, and partially reconstitute the adaptive immune system [4]. Consequently, greater number of infected individuals live longer by overcoming the health-related consequences and challenges associated with HIV [5], hence increasing the pool of infected people. This, in turn adds to the societal burden of the disease [6].

Apart from improving the health of HIV infected persons, HAART adversely affects their health. Such adverse effects, among arrays of complications, include changes in lipid profile, just as the HIV infection itself does [7]. For instance, a condition known as lipodystrophy emerged post HAART era [7]. Lipodystrophy is associated with physical and metabolic changes whereby the body finds it difficult to produce, utilize and distribute fat with resultant alteration in body composition [4]. This alteration in body composition results to accumulation and reduction of fat in some parts of the body. These morphological changes also augment alteration in lipid profile [4]. Scevola et al. [8] reported that lipodystrophy is associated with elevated triglycerides and low-density lipoprotein, which may place an individual at risk of cardiovascular diseases (CVDs). It is estimated that 20–60% of persons living with HIV and receiving HAART experience glucose and/or lipid profile irregularities [9]. Hence, the collective effects of these multiple metabolic abnormalities represent a growing concern for increased risk of diabetes mellitus, renal and cardiovascular diseases. With this gamut of problems experienced by HIV population, Tiozzo et al. [4] advocated healthy lifestyle changes for them, with special emphases on regular exercise and healthy diets.

Several studies [4,5,10–13] had demonstrated the efficacy of exercise on physiological and immunological functions of HIV infected persons. Either aerobic or resistance exercise, or both are of utmost benefits to this population, without the gratuitous cost, toxicity and potential adverse effects of pharmacological interventions [14]. However, previous studies [5,15,16] had examined the benefits of these two forms of exercise in HIV population with aerobic exercise being more widely used. Veljkovic et al. [17] reported that aerobic exercise acts as an immune stimulant for both HIV positive and HIV negative individuals by creating a type of natural vaccine. These scholars asserted that aerobic exercise if widely adopted will contribute to worldwide reduction of HIV pandemic and probably leads to its prevention.

However, apart from the observed poor attitude towards exercise among HIV population, and indeed the general public, fatigue, one of the major reported symptoms of HIV infected persons, prevents them from engaging in any form of exercise. Consequently, in addition to their low immunity, they present with reduced functional capacity and metabolic syndrome [18]. This inability to engage in exercise also has the potential to exacerbate HIV-related symptoms and accelerate the rate of disease progression to acquired immunodeficiency syndrome (AIDS). Thus, engaging in exercise at moderate intensity levels is of utmost importance for HIV population. In addition, in most developing countries, including Nigeria, little or no attention is paid to the benefits of exercise in the overall management of HIV infection and complications arising from its drug therapy.

The authors therefore envisaged that this little or no attention paid to the efficacy of exercise on the health of HIV infected persons in Nigeria may be the basis for the lack of literature on the effect of exercise on the health of the overwhelming population living with HIV and AIDS in the country. Probably, this may be the rationale why the authors have observed over the years, the non inclusion of therapeutic exercise as one of the treatment strategies for the vast number of HIV populations attending antiretroviral (ARV) clinic at the tertiary hospital where this study was conducted. The failure to include exercise in the management of HIV infected persons may be as a result of lack of very good knowledge on effect of exercise on HIV population among substantial number of health care professionals as reported by Maduagwu et al. [19]. These observation and report, as well as paucity of literature in Nigeria on this field prompted the authors to conceptualize this study.

## Materials and Methods

### Participants

Population for this study comprised HIV infected persons attending ARV clinic at a tertiary hospital in Northeastern Nigeria. The choice of this hospital was based on the fact that it has an established ARV clinic that provides care to about 6,000 HIV infected persons, and AIDS associated infections every month. Volunteer and willing 91 (56 females and 35 males) HIV infected persons enrolled in the study. However, 82 (49 females and 33 males) met the inclusion criteria, and were eligible to participate. These 82 participants were free from AIDS, 20 years or older and were on ARV therapy consistently for not less than six months. Participants excluded from this study were those with the following attributes: significant cognitive impairment or inability to follow instructions; current or past use of hormone therapy; involvement in a regular exercise program (defined as two or more structured exercise sessions weekly for more than or equal to six months prior to enrolment); respiratory and neurologic conditions, e.g. asthma, neuropathies, stroke etc. Pregnant women and breast feeding mothers were also excluded.

### Design

Randomized control group experimental design was employed to assign the 82 participants to experimental or control group. This random assignment involved the authors writing “1” on each 41 small pieces of papers and “2” on another 41 small pieces of papers. The 82 papers were then wrapped, placed and mixed in a basket. Participants that picked papers with “1” written on them were assigned to control group while those that picked papers with “2” were allocated to experimental group. At the end of this allocation, control (23 females and 18 males) and experimental (26 females and 15 males) groups had 41 participants each. However, at the end of the study, 22% attrition rate was recorded generally, with each group having 32 participants; control group (19 females and 13 males) and experimental group (22 females and 10 male).

This high attrition rate was as a result of relocation of some participants due to the insurgency in the Northeast, Nigeria during the period of study. Four participants dropped from the experimental group because the authors were unable to meet up with their demands for burdensome transport fares and settlement for other medical bills not related to the study. Three participants (two in the experimental group and one in the control group) were advised to withdraw from the study because of their inconsistent attendance to the program. Each participant had to make 100% attendance to be included in data analysis. HIV infected persons attending ARV clinic at the tertiary hospital already have identification numbers issued by the clinic. The authors used these identification numbers to recognize and identify the participants throughout the period of this study.

### Procedure

The authors explained the objectives, protocols and benefits of the study to the participants. The participants signed or thumb printed informed consent forms. For the participants who neither read nor understand English Language, Hausa Language translated version was made available. All participants understand Hausa Language, the principal local language in Northern Nigeria. Ethical approval was obtained from the Research and Ethical Committee of the tertiary hospital where the study was conducted. The principal author sat for and passed Biomedical Research Basic/Refresher (Reference ID: 11844307) course in order to satisfy Collaborative Institutional Training Initiative (CITI) requirements for researchers involved primarily in biomedical research with humans. Two research physicians at the ARV clinic of the hospital screened the participants for eligibility based on the inclusion/exclusion criteria and conducted physical examination on each participant. Each participant in addition to the screening and physical examination filled a health status screening form. Participants filled the bio-data forms which comprised sociodemographic and socioeconomic status information. Two laboratory scientists from different specialties of laboratory medicine collected blood samples from the participants. The one from Immunology Department collected the blood samples with tripotassium ethylene diaminetetracetic acid (K_3_EDTA) bottles, and analyzed CD4 cell counts, the other from Chemical Pathology used plain bottles for lipid profile. These evaluations were performed at baseline and after the 12th week of the aerobic exercise training by the same medical laboratory scientists in order to minimize error and ensure reliability. The blood samples were collected from the participants in the morning following an overnight fast of 10–12 hours.

### Intervention/Treatment

The experimental group participated in 12 weeks moderate intensity treadmill aerobic exercise, three times per week for 40 minutes (5 minutes warm up phase, 30 minutes conditioning/training phase and 5 minutes cool down phase) per session. The exercise intensity was between 50 and 75% of heart rate reserve (HRR). These chosen parameters (mode, frequency, duration and intensity) for this study were in line with those used in previous studies [4,20,21].

The training protocol is shown in table 1. The target training heart rate (TTHR)/training intensity was pegged at 50% of HRR from 1st week– 2nd week and increased to 75% at the 11th - 12th week. The Karvonen method was used to determine the TTHR for the participants. Calitz [22] reported that most HIV studies use this method to determine the TTHR of HIV population. The aerobic exercise training was performed on computerized treadmills which are equipped with the necessary parameters, such as speed per hour, distance covered per time, inclination etc. The inclination of the treadmills was at 0° to reduce the effect of the stress of exercise on the participants because of their HIV status which predisposes them to early fatigue. Digital heart rate monitor (Port Washington, New York, USA) was used to observe the target training heart rate of each participant.

**Karvonen equation:** TTHR = {Heart Rate Reserve (HRR) x exercise intensity} + HR_rest_. HRR = HR_max_ – HR_rest_; where, HR_rest_ = Resting heart rate; HR_max_ = 220 – age.

The participants in the control group were implored not to participate in any form of exercise apart from their routine daily activities throughout the period of study. The principal author telephoned them every 3 weeks to maintain contact and promote their interest in the study. Also, the authors organized lectures for this group once a week on issues pertaining to nutrition, meaning of HIV and AIDS, relationship and differences between HIV and AIDS, mode of HIV transmission and its prevention, and adherence to ARV therapy.

### Data analysis

Descriptive statistics of percentages and frequency counts summarized socio-demographic characteristics of the participants. Paired Student t-test compared significant difference between the variables at baseline and 12th week in the experimental group, and also in the control group. Student t-test for independent samples analyzed significant difference in the variables between the control and experimental groups at baseline and the end of the study. All analyses were executed using Statistical Package for the Social Sciences (SPSS) version 17.0 software (SPSS Inc., Chicago, Illinois, USA). P< 0.05 was considered significant.

## Results

Table 2 displays the socio-demographic characteristics of the participants. Mean ages in years of the 64 participants, the control and experimental groups were 39.57 ± 10.13, 39.38 ± 10.03 and 40.84 ± 10.05 respectively. Their respective age ranges in years were 21–60, 21–60 and 20–60. Females, married participants and civil servants were in majority. Participants with tertiary education were in preponderance in the control group, while in the experimental group, those who had no formal education predominated. Participants without any means of income and those that earn less than N41, 000.00 formed the majority in both groups. Table 3 shows that there was insignificant difference (p > 0.05) at baseline in the variables between the experimental and control groups. Table 4 presents the pre – and post – test values of the variables in the experimental and control groups. Table 5 depicts post – test comparison of variables between the participants in the control and experimental groups.

## Discussion

Effect of moderate intensity aerobic exercise on CD4 cell counts, total cholesterol (TChol), triglyceride (TG), high density lipoprotein (HDL) and low density lipoprotein (LDL) of HIV infected persons was examined. At baseline, unpaired Student t-test showed insignificant difference in the variables between the experimental and control groups. This implies baseline similarities of the two groups; hence observed changes at the end of this study could be attributed to the effect of the aerobic exercise training.

The result indicated significant improvement in the variables between pretest and post-test in the experimental group. Significant difference also existed in these variables between the experimental and control groups at the end of the study. In the control group, there was significant improvement and significant increase in CD4 cell counts and TG respectively between pre- and post- tests. TChol, HDL and LDL showed insignificant difference. The significant improvement in CD4 cell counts found in the experimental group agrees with the findings of some past studies, and relatively at variance with others which reported stability or insignificant improvements. Terry et al. [23] reported insignificant increase in CD4 cell counts of HIV infected persons after 12 weeks aerobic exercise. However, other studies [13,24–26] reported an increase in CD4 cell counts, while few [20,27] found stability in CD4 cells. Tiozzo et al. [4] in their study demonstrated more stable CD4 cell counts from baseline of −3% in the experimental participants, while the control group experienced significant reduction of −16% after 12 weeks.

Ezema and colleagues [13] reported significant increase of 27.18% in CD4 cell counts in the experimental group, while the control group showed insignificant increase of 3.60%. This present finding indicated 36.34% and 8.04% increase in CD4 cell counts in the experimental and control groups respectively, conforming to that of Ezema et al. [13]. Mustafa et al. [28] observed that HIV infected individuals’ self-reporting exercise participation had 107.5% higher CD4 cell counts compared to HIV infected non exercise participants. Exercisers also displayed slower disease progression to AIDS, less symptoms and decreased rates of mortality compared to non exercisers. MacArthur et al. [29] linked lower CD4 cell counts to noncompliance with prescribed exercise.

This significant improvement in CD4 cell counts in the experimental group could be attributed somehow to the lower scores observed in baseline quality of life domains of the participants which improved significantly at the end of the study. This article is a part of a comprehensive study which assessed among other variables, the participants’ quality of life. Anxiety and depression are some of the most common symptoms experienced by HIV population and indicators of low quality of life with concomitant stress [30]. Stress reduces CD4 cell counts [25], while exercise enhances CD4 cell counts by reducing negative emotional states and modulating levels of endogenous opiates and stress hormones [31]. This remarkable increase in CD4 cell counts could also be attributed to the fact that aerobic exercise training stimulates the formation of certain antibodies that prevent some potent HIV protein molecules (glycoprotein {gp} 120) from attaching to receptor sites of CD4 cells [32]. By this process, the damaged immune system is reconstituted, and HIV disease progression to AIDS is slowed down [33], similar to the role played by HAART.

The significant improvement in CD4 cell counts found among the participants in the control group at the end of the study may be confounding. The control group did not participate in the exercise training, rather the authors organized weekly lectures for them on nutrition and the importance of adherence to antiretroviral (ARV) drugs among others. It could be that these participants applied what they learnt from these organized lectures. Good nutrition improves immune health, and ARV therapy reconstitutes immune system [4].

This study found significant improvement in lipid profile of participants in the experimental group. This agrees with that of Thoni et al. [34] which reported significant improvement in TChol, TG and HDL. Thoni and colleagues [34] reported 23% and 43% reduction in TChol and TG respectively, and 6% increase in HDL among 17 lipodystrophic and dyslipidemic HIV infected persons after 16 weeks aerobic exercise training. The result of this present study indicated 28.1%, 37. 9% and 44.4% reduction in TChol, TG and LDL respectively and 79.7% increase in HDL among 32 HIV infected persons who completed the 12 weeks aerobic training program. These higher percentages recorded in this study when compared to those reported by Thoni et al. [34 could be as a result of differences in sample size, the gap in years of study and population. This finding is also somewhat similar to that of Halbert et al. [35] which showed favorable changes in lipids after 12 weeks of exercise training in HIV infected persons with dyslipidemia who were not on ART. However, Halbert and colleagues [35] did not report whether this favorable changes were significant or not, and their participants were not on ART, unlike this present study which enrolled participants on HAART and the changes in lipid profile were significant.

Contrary to the finding of this study, Birk et al. [36] found insignificant reduction in TG after 12 months of aerobic exercise among HIV infected population. Also, Terry et al.16 reported insignificant improvements in TG, TChol and HDL after 12 weeks of aerobic exercise among HIV infected individuals receiving ART. Furthermore, Tiozzo et al. [4] found insignificant change in lipid profile of their participants after 12 weeks of exercise training. Tiozzo and colleagues [4] posited that the lack of changes in lipid profile may be related to the fact that the participants were not dyslipidemic at baseline. Normal levels of these biochemical substances are as follows: TChol (<200mg/dl); TG (<160mg/dl); HDL (40 – 59mg/dl); LDL (<100mg/ dl) [37]. In this present study, the mean values of these substances at baseline among the participants in the experimental group were TChol (217.33±54.52), TG (182.52±97.46), HDL (32.10±15.08) and LDL (131.48±48.72). Based on these values, we are of the opinion that the participants in our study presented with dyslipidemia at baseline as reported by the aforementioned two similar studies [34,35], hence the significant improvement observed at the end of the study. Control group also presented with insignificant changes in lipid profile, except for TG at the end of the study. Since the baseline lipid profiles in both groups were similar, it could also be inferred that the control group were dyslipidemic at baseline.

## Limitations

The rate of attrition recorded during the period of this study might have affected the results since we could not control this in anyway. This occurrence might have introduced selection bias in the study.

## Conclusion

Twelve weeks aerobic exercise training improved CD4 cell counts and lipid profile of HIV infected persons. Emphasis on good nutrition and adherence to antiretroviral therapy by health care providers and/or exercise scientists may enhance immune function in HIV population.

## Figures and Tables

**Table 1:** Summary of Training Protocol for Experimental participants.

Week	Mode	Frequency	Duration Intensity*	*Speed (km/h)	
2-Jan	treadmill	3	5/30/2005	50% of HRR	1.5
4-Mar	treadmill	3	5/30/2005	55% of HRR	2
6-May	treadmill	3	5/30/2005	60% of HRR	2.5
8-Jul	treadmill	3	5/30/2005	65% of HRR	3
10-Sep	treadmill	3	5/30/2005	70% of HRR	3.5
12-Nov	treadmill	3	5/30/2005	75% of HRR	4

* Starting speed; was being adjusted until the required intensity was reached. HRR; Heart Rate Reserve (HR_max_ − HR_rest_); Intensity = target training heart rate/training.

**Table 2:** Socio-demographic Characteristics of the Participants (n = 64).

Variables	Control group 12thwk n = 32	Experimental group 12thwk n = 32
**Age** (years)	39.38 ± 10.03 (21–60)*	40.84 ± 10.05 (20–60)*
**Age** (years; n = 64)	39.57 ± 10.13 (21–60)*	
**Gender**		
Male	13 (40.6)	10 (31.2)
Female	19 (59.4)	22 (68.8)
**Marital status**		
Single	7 (21.9)	3 (9.4)
Married	20 (62.5)	22 (68.7)
Divorced/separated	4 (12.5)	2 (6.3)
Widowed	1 (3.1)	5 (15.6)
**Educational level**		
None	5 (15.6)	14 (43.8)
Primary	8 (25.0)	6 (18.7)
Secondary	8 (25.0)	4 (12.5)
Tertiary	11 (34.4)	8 (25.0)
**Occupation**		
Civil servant	18 (56.2)	17 (53.0)
Business	5 (15.6)	2 (6.3
Artisan	3 (9.4)	3 (9.4)
Unemployed	3 (9.4)	4 (12.5)
House wife	3 (9.4)	4 (12.5)
Others	0 (0.0)	2 (6.3)
**Monthly income**		
None	6 (18.8)	8 (25.0)
≤20,000	7 (21.9)	10 (31.3)
21,000–40,000	8 (25.0)	9 (28.1)
41,000–60,000	4 (12.5)	1 (3.1)
61,000–80,000	2 (6.2)	1 (3.1)
80,000– 100,000	2 (6.2)	0 (0.0)
>100,000	3 (9.4)	3 (9.4)

**Key:** (21–60)^*^ = age ranges of the participants in the control group;. (20–60)^*^ = age range of the participants in the experimental group; (21 – 60)^*^= age range of the 64 participants

**Table 3:** Independent Student t-test on mean baseline CD4 cell count and lipid profile of the control and experimental groups.

Variables	Control group	Exp. group	t-test	p-value
CD4 (cells/mm^3^)	483.76 ± 135.99	493.07 ± 144.51	0.301	
T-Chol (mg/dl)	213.46 ± 55.30	217.33 ± 54.52	0.327	0.745
Triglyceride **(**mg/dl)	194.92 ± 82.40	182.52 ± 97.46	0.616	0.54
HDL **(**mg/dl)	32.48 ± 11.60	32.10 ± 15.08	0.095	0.925
LDL **(**mg/dl)	133.02 ± 45.63	131.48 ± 48.72	0.127	0.899

**Table 4:** Summary of paired Student t-test analysis comparing the baseline and 12th week mean values of CD4 cell counts and lipid profile in the experimental and control groups.

Variables	Pre-test n=32	Post-test n=32	Df 31	t-test	p-value
**Experimental** **group**			6.157		
CD4 (cell/mm^3^)	491.18 ± 152.73	672.25 ± 227.59		0.000*	0
T-Chol (mg/dl)	214.56 ± 52.49	156.23± 35.58		10.557	0
Triglyceride (mg/dl)	180.36 ± 93.23	113.41 ± 60.25		6.928	0
HDL (mg/dl)	35.86 ± 12.04	57.67 ± 47.95		4.584	0
LDL (mg/dl)	128.07 ± 65.71	73.09 ± 25.91		8.019	0
**Control groip**					
CD4 (cell/m^3^)	489.53 ± 108.78	522.66 ± ± 123.06		3.837	0.001*
T-Chol (mg/dl)	222.84 ± 47.51	224.29 ± 52.98		1.802	0.121
Triglyceride (mg/dl)	198.47 ± 62.21	207.32 ± 80.63		3.432	0.014*
HDL (mg/dl)	31.13 ± 10.27	29.78 ± 9.67		1.579	0.537
LDL (mg/dl)	134.16 ± 42.76	135.73 ± 39.44		0.948	0.592

* Shows where significant difference exists, that is, p < 0.05

**Table 5:** Independent Student t-test on mean 12th week values of CD4 cell count and lipid profile of the control and experimental groups.

Variables	Control group N=32	Exp. Group N=32	t-test value	p-value
CD4 (cells/mm^3^)	522.66 ± 123.06	672.25 ± 227.59	3.271	0.002*
T-Chol	224.29 ± 52.98	156.23 ± 35.58	6.012	0.000*
Triglyceride	207.32 ± 80.63	113.41 ± 60.25	5.27	0.000*
HDL	29.78 ± 9.67	57.67 ± 47.95	4.481	0.000*
LDL	135.73 ± 39.44	73.09 ± 25.91	7.544	0.000*

* Shows where significant difference exists, that is, p < 0.05
